# Advanced Imaging and Occupational History in the Diagnosis of Bird Fancier's Lung: A Case Report

**DOI:** 10.7759/cureus.77522

**Published:** 2025-01-16

**Authors:** Louay Kila, Junaid Sheikh, Brian Casserly, Sarah Hazri, Irfan Amin

**Affiliations:** 1 Respiratory Medicine, University Hospital Limerick, Limerick, IRL

**Keywords:** bird fancier's lung, covid-19, high-resolution computed tomography, hypersensitivity pneumonitis, occupational history

## Abstract

Bird fancier's lung (BFL) is a subtype of hypersensitivity pneumonitis (HP), an immune-mediated interstitial lung disease (ILD) resulting from the repeated inhalation of avian proteins found in bird droppings, feathers, and serum. Diagnosing BFL is challenging due to nonspecific symptoms that overlap with other ILDs like idiopathic pulmonary fibrosis and sarcoidosis. This complexity is heightened during pandemics such as coronavirus disease 2019 (COVID-19), where respiratory symptoms may be misattributed to severe acute respiratory syndrome coronavirus 2 (SARS-CoV-2) infection, leading to diagnostic anchoring and delays in appropriate management. High-resolution computed tomography (HRCT) is pivotal in detecting subtle pulmonary changes, characteristic of HP, surpassing standard chest radiographs. We present the case of a 43-year-old male pigeon keeper with an eight-week history of progressive dyspnea on exertion and intermittent chest pain. Despite unremarkable chest X-rays, HRCT revealed bilateral diffuse centrilobular nodules, patchy ground-glass opacities, and a mosaic attenuation pattern without fibrosis, consistent with acute HP. A thorough occupational history uncovered significant avian antigen exposure, and a family history suggested genetic susceptibility. The patient was diagnosed with BFL and treated with a tapering regimen of oral corticosteroids, starting at 40 mg/day. He was advised to cease pigeon keeping and avoid future avian exposure. Significant symptomatic improvement occurred within three months. Follow-up imaging over one year confirmed stable lung parenchyma with no disease progression or recurrence. This case underscores the importance of incorporating detailed occupational histories and utilizing advanced imaging modalities like HRCT when standard imaging is inconclusive. Early identification and intervention are crucial to prevent progression to chronic HP and irreversible fibrosis. Management should focus on reducing inflammation with corticosteroids and implementing strict environmental controls to prevent re-exposure. Long-term follow-up is essential to monitor for recurrence and maintain remission. Clinicians should remain vigilant for alternative diagnoses during pandemics to avoid diagnostic anchoring. This case contributes to the evidence supporting HRCT's critical role in early HP detection and emphasizes heightened clinical awareness of occupational lung diseases. A multidisciplinary approach involving pulmonologists, radiologists, and occupational medicine specialists is key to optimizing outcomes in HP and other ILDs.

## Introduction

Hypersensitivity pneumonitis (HP) is an immune-mediated interstitial lung disease (ILD) characterized by an exaggerated immune response to inhaled environmental antigens in susceptible individuals [1]. Bird fancier's lung (BFL) is a subtype of HP resulting from exposure to avian proteins found in bird droppings, feathers, and serum [2]. Individuals frequently exposed to avian antigens, such as pigeon keepers, poultry farmers, and pet bird owners, are at an increased risk of developing BFL due to prolonged exposure [3].

Diagnosis of BFL is problematic, as its clinical presentation, which includes symptoms such as cough, dyspnea, and chest discomfort, may overlap with other ILDs like idiopathic pulmonary fibrosis and sarcoidosis, making differential diagnosis difficult [4,5]. Conventional chest radiographs often fail to detect early or subtle interstitial changes, leading to delayed diagnosis [6]. On the other hand, high-resolution computed tomography (HRCT) has turned out to be an essential imaging technique in the demonstration of typical radiographic features in HP such as ground-glass opacities, centrilobular nodules, and mosaic attenuation patterns [7].

The diagnosis of BFL relies on the establishment of exposure to avian antigens, compatible clinical and radiological findings, and exclusion of other ILD causes. Unlike other forms of HP, such as farmer's lung or humidifier lung, BFL involves exposure to avian proteins, and recurrent or prolonged exposure increases the risk of chronic HP progression [4,5]. Clinically, BFL differs from idiopathic pulmonary fibrosis by its association with antigen exposure, non-honeycombing fibrosis on HRCT, and the absence of features such as clubbing in the early stages [8].

The coronavirus disease 2019 (COVID-19) pandemic has further complicated the diagnostic process for respiratory diseases. The high prevalence of COVID-19 created a diagnostic bias, with anchoring in the common respiratory symptoms often being misattributed to severe acute respiratory syndrome coronavirus 2 (SARS-CoV-2) infection, delaying the diagnosis of other respiratory conditions such as BFL [8]. This illustrates the importance of keeping a broad differential diagnosis, especially for patients with substantial occupational or environmental exposures.

Early recognition and intervention in BFL are important to avoid the progression to chronic HP, which may result in irreversible pulmonary fibrosis [9]. The management strategies for BFL mainly include antigen avoidance, reduction of exposure to avian proteins, and the use of corticosteroid therapy for the control of inflammation [10]. HRCT, along with an appropriate occupational history, is an important modality for the early diagnosis and management of BFL [11]. This timely diagnosis can permit clinicians to intervene before the onset of chronic, irreversible damage [12].

This case report showcases the importance of a detailed occupational history coupled with advanced imaging in the diagnosis of BFL during the COVID-19 pandemic, a period when diagnostic biases may occur. We present a case in which standard imaging initially seemed unremarkable; however, HRCT and a good occupational history led to the appropriate diagnosis and management, with good patient outcomes.

## Case presentation

A 43-year-old male pigeon fancier was referred to the emergency department in June 2020 for a progressive eight-week history of dyspnea on exertion and episodes of chest tightness. Further history revealed that he had restarted his lifelong hobby of pigeon keeping three months prior to the onset of his symptoms. His past medical history was unremarkable, and he was noted to be an ex-smoker with a 15-pack-year history. His brother had also developed a lung condition associated with similar avian exposure.

On admission, the patient's vital signs were stable. Physical examination revealed clear lung fields bilaterally with normal heart sounds. Initial laboratory tests showed an elevated C-reactive protein (CRP) of 37 mg/L, a normal white blood cell (WBC) count of 7.83 × 10^9^/L, and a total immunoglobulin E (IgE) level of 5.4 kIU/L within normal limits (Table 1). An electrocardiogram (ECG) and serial troponins were normal, and a SARS-CoV-2 polymerase chain reaction (PCR) test was negative.

**Table 1:** Laboratory investigation results. CRP: C-reactive protein; WBC: white blood cell; Hb: hemoglobin; LDH: lactate dehydrogenase; IgE: immunoglobulin E

Parameter	Obtained value	Reference range
CRP	37 mg/L	<5 mg/L
WBC count	7.83 × 10⁹/L	4.0-11.0 × 10⁹/L
Hb	14.3 g/dL	13.0-17.0 g/dL
Platelets	235 × 10⁹/L	150-450 × 10⁹/L
LDH	180 U/L	140-280 U/L
Total IgE	5.4 kIU/L	<114 kIU/L

**Figure 1:**
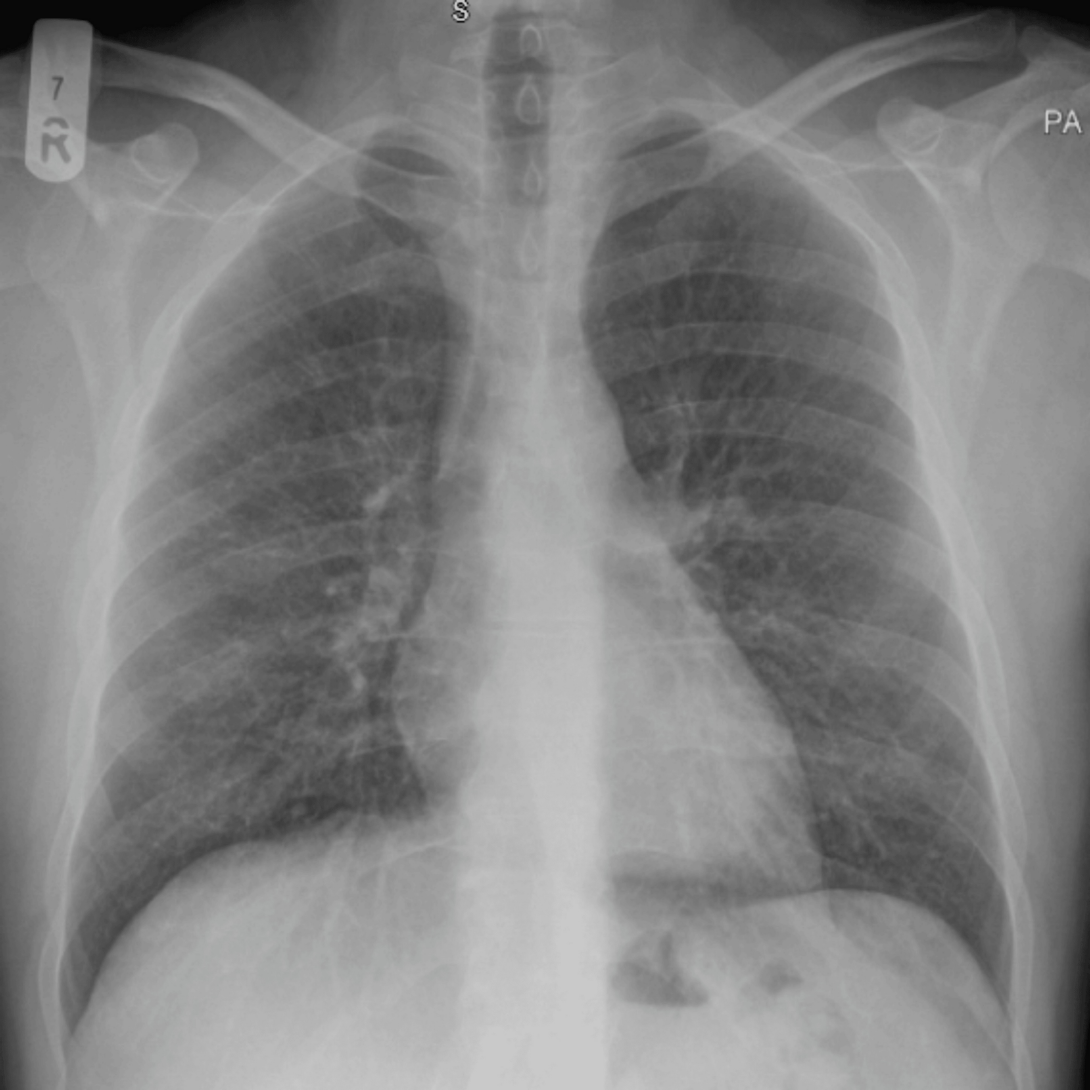
Initial chest X-ray on June 9, 2020, showing clear lung fields with no signs of pneumothorax or consolidation. PA: posterior-anterior

A chest X-ray was performed on June 9, 2020, which showed clear lungs bilaterally, no focal area of consolidation, and a cardiomediastinal silhouette within normal limits, with no signs of pneumothorax (Figure 1).

**Figure 2:**
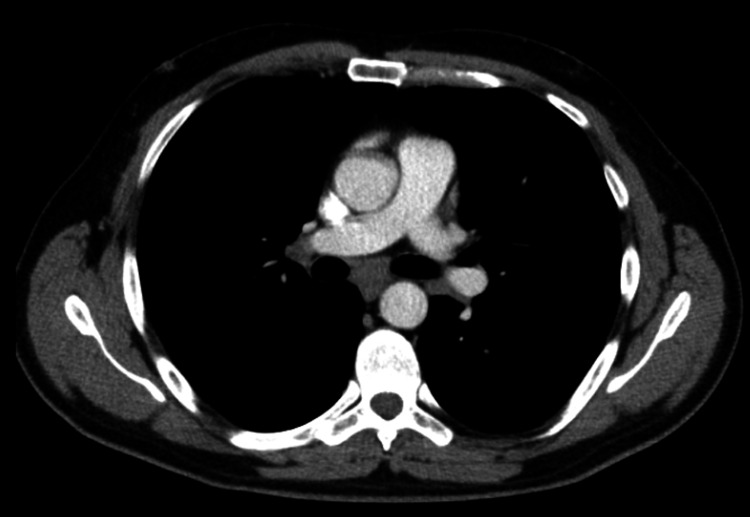
Subcentimeter mediastinal and bilateral hilar lymph nodes.

**Figure 3:**
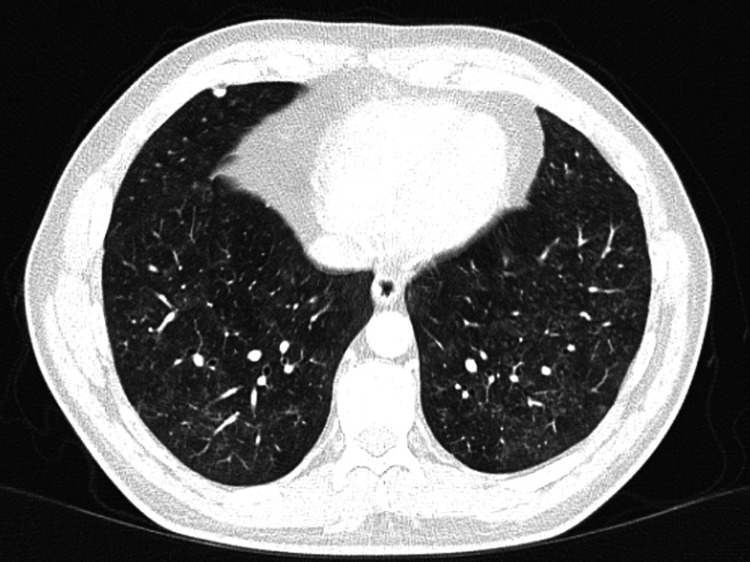
Mosaic attenuation pattern and calcified granulomata, with the largest measuring 6 mm.

Despite these normal findings, clinical suspicion for BFL remained high. Consequently, an HRCT of the thorax was ordered on June 10, 2020. HRCT findings included bilateral diffuse centrilobular nodules, patchy ground-glass opacities, a mosaic attenuation pattern, and no interstitial septal thickening or fibrotic changes (Figure 2 and Figure 3). Few calcified pulmonary granulomata were noted, with the largest measuring 6 mm in the right middle lobe. These findings were consistent with acute HP, specifically BFL. The differential diagnosis included chronic HP, idiopathic pulmonary fibrosis, and sarcoidosis. However, the absence of significant fibrotic changes and the patient's detailed occupational history pointed towards an acute form of BFL [5].

Following the HRCT findings, the patient was diagnosed with BFL. He was started on a tapering dose of oral corticosteroids (prednisolone, starting at 40 mg/day). Additionally, Spiriva Respimat and Ventolin PRN were prescribed to manage symptoms. Crucially, the patient was advised to remove the pigeons from his household and avoid future exposure to avian antigens. The importance of strict adherence to environmental control measures was emphasized to prevent disease recurrence.

Follow-up

**Figure 4:**
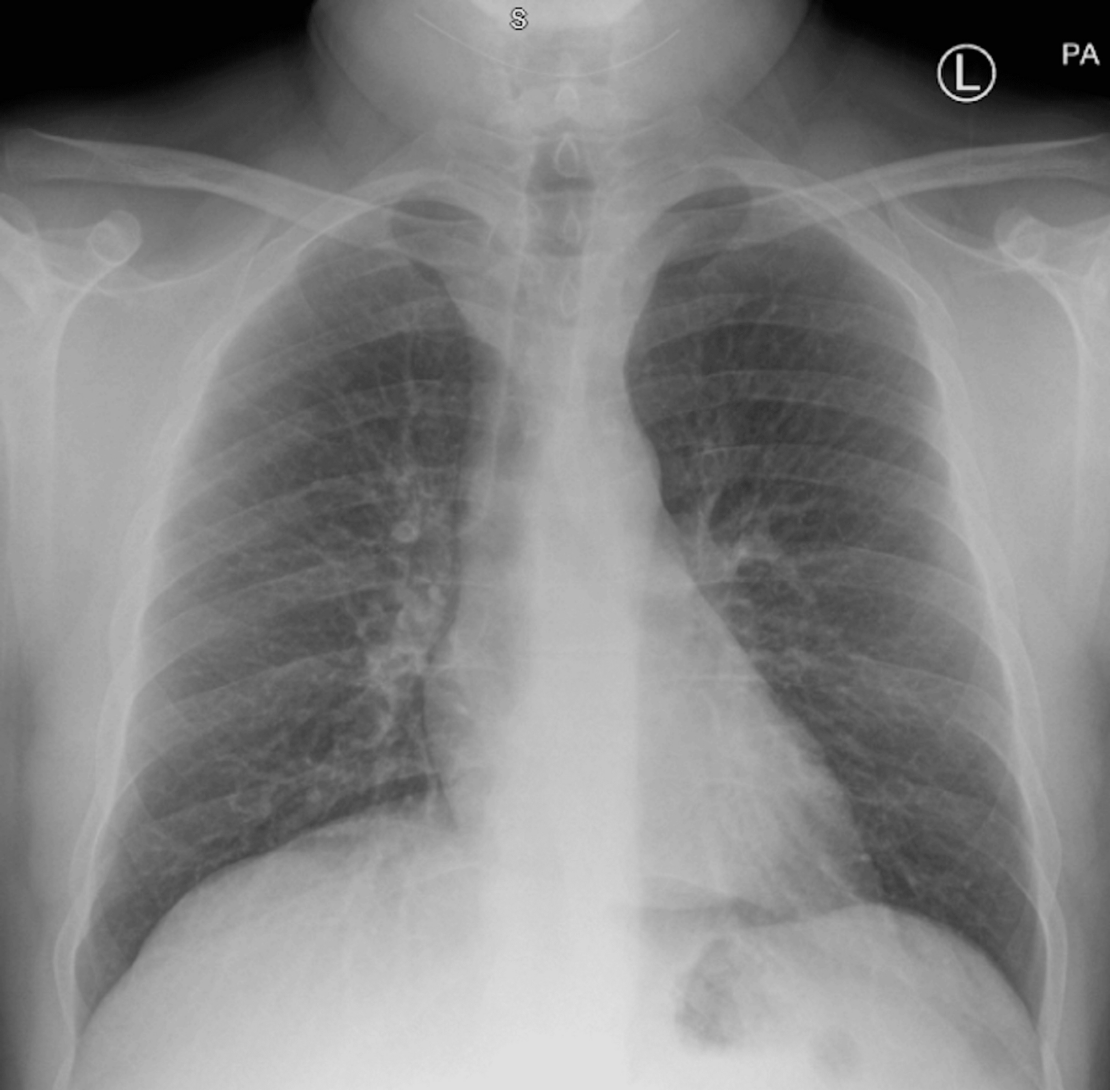
Follow-up chest X-ray in December 2020 showing stable mediastinal silhouettes and no interval onset of new findings. PA: posterior-anterior

**Figure 5:**
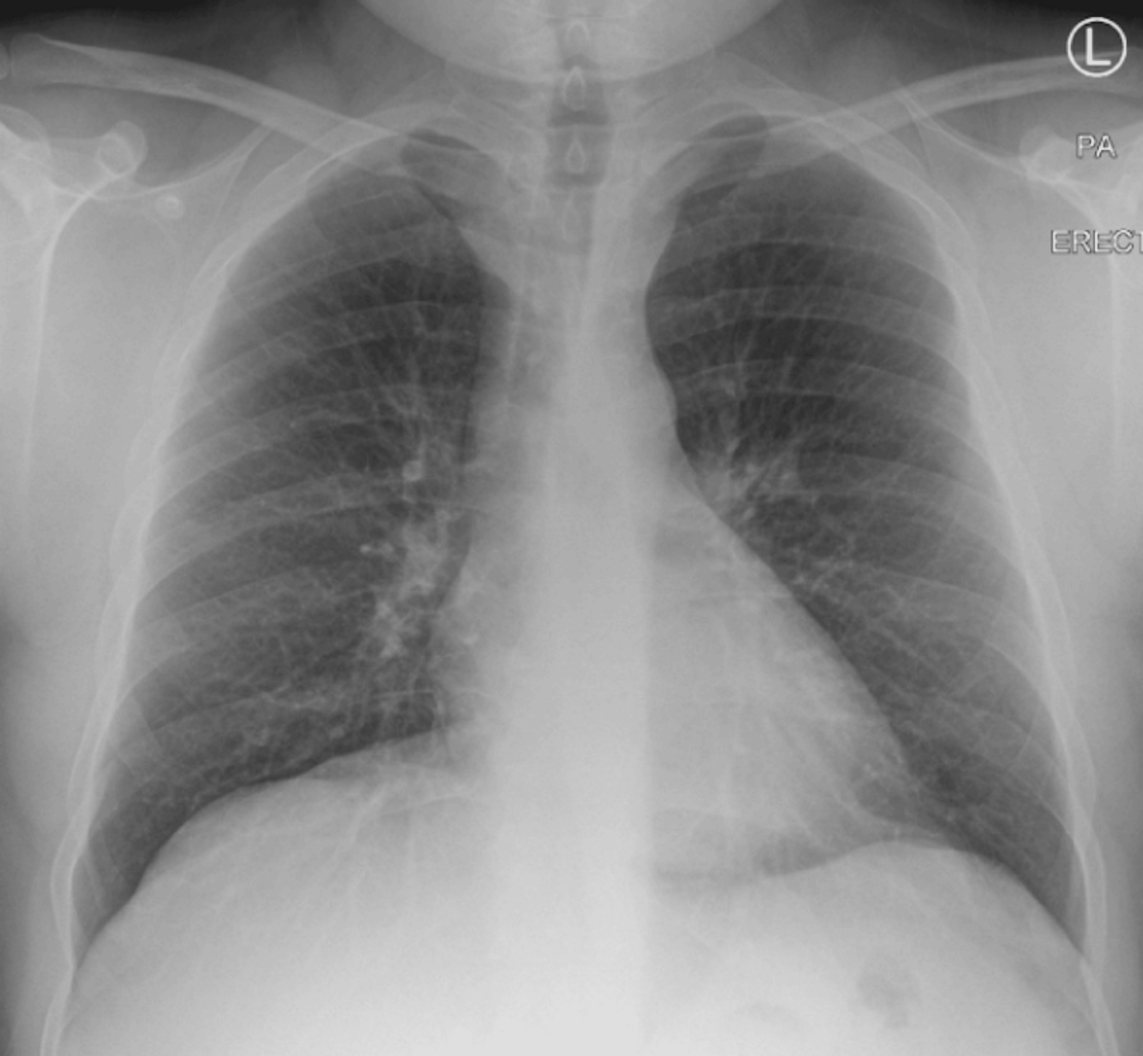
Follow-up chest X-ray in May 2021 showing stable mediastinal silhouettes and no interval onset of new findings. PA: posterior-anterior

**Figure 6:**
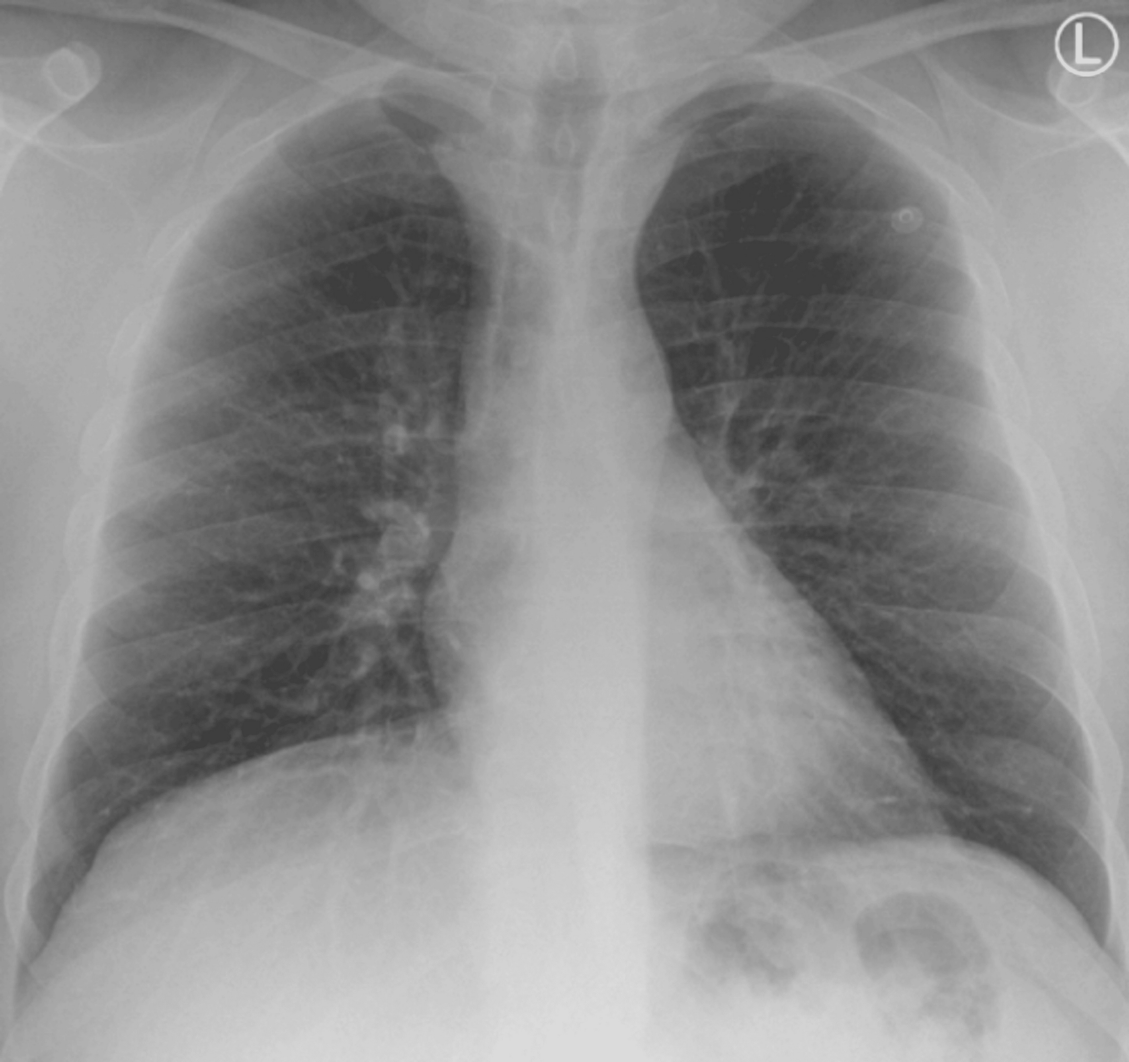
Follow-up chest X-ray in February 2024 showing stable mediastinal silhouettes and no interval onset of new findings.

At the three-month follow-up, the patient reported significant improvement in symptoms, with no episodes of dyspnea or chest pain. Subsequent repeat chest X-rays in December 2020, May 2021, and February 2024 confirmed no new pathological changes (Figure 4, Figure 5, and Figure 6). Heart size remained within normal limits, and the mediastinal silhouette remained stable compared to prior examinations. A calcified right midzone pulmonary nodule typical for granuloma was noted, with no interval onset of consolidation or pleural effusion. These findings confirmed the stability of the patient's condition and the effectiveness of the management strategies, including corticosteroid therapy and strict environmental control. At one year post-diagnosis, the patient remained symptom-free with no signs of recurrence on clinical examination or follow-up imaging [12].

 Written informed consent was obtained from the patient for the publication of this case report and any accompanying images.

## Discussion

COVID-19 and diagnostic anchoring

During the COVID-19 pandemic, many clinicians anchored respiratory symptoms to SARS-CoV-2 infection, delaying the consideration of other diagnoses. The high prevalence of COVID-19 created a diagnostic bias, especially when patients presented with typical symptoms of dyspnea and chest pain. This diagnostic anchoring bias persisted despite multiple negative PCR tests for COVID-19, leading to delays in identifying other causes of respiratory symptoms, such as BFL [7]. Diagnostic guidelines developed by the American Thoracic Society/European Respiratory Society (ATS/ERS) provide an essential framework for differentiating ILDs, hence highlighting the importance of thorough diagnostic workups to avoid COVID-19 being used as a sole diagnosis in patients with potential ILDs [13].

Pathophysiology of HP

HP is an immune-mediated lung disease induced by the inhalation of a variety of organic antigens, such as avian proteins in the present case. The main cell types involved in HP immune response are CD4+ and CD8+ T cells, with prevalent Th1 and Th17 responses. These immune cells will recruit neutrophils and lymphocytes to the lung interstitium, leading to inflammation and, in chronic cases, fibrosis [1]. Despite the high frequency of pigeon keepers exposed to the antigens, only some develop HP, indicating thereby that genetic susceptibility and some environmental factors may play a role [2,14]. Moreover, the type and intensity of antigen exposure are not the only influencing factors in the development of HP, and an individual's immune response and genetic makeup also come into play, as has been pointed out by guidelines emphasizing that recognizing environmental and genetic interactions in HP diagnosis is paramount [15]. In this case, the suspicion of BFL was raised by a history of prolonged exposure to pigeons, but again, mere exposure was not sufficient for confirming HP. HRCT and serological tests, including precipitating antibodies to avian antigens, were needed to confirm HP among other ILDs [5].

Exposure and hypersensitivity

Established risk factors for developing HP include exposure to avian antigens; however, not everyone exposed develops the disease, which indicates the complexity of its pathophysiology. The antigen load, genetic predisposition, and immune responses will affect the likelihood of disease onset. The most common antigens in BFL are derived from bird droppings, feathers, and serum [3]. However, patients usually continue to be exposed to these antigens until symptoms get worse. In this case, the patient resumed pigeon keeping shortly before his symptoms worsened, underlining the importance of taking a detailed environmental and occupational history in patients presenting with unexplained respiratory symptoms [4].

Role of HRCT in diagnosis

HRCT is crucial in diagnosing ILDs by providing detailed imaging of the lung parenchyma. In HP, HRCT typically reveals centrilobular nodules, ground-glass opacities, and mosaic attenuation, all of which were present in this patient's scan [6]. These imaging findings are crucial, particularly when chest X-rays appear normal, as in this case. A study comparing chest X-rays with HRCT in ILD diagnosis found that chest X-rays missed interstitial abnormalities in up to 40% of cases, while HRCT demonstrated much higher sensitivity and specificity [8]. HRCT's ability to detect subtle parenchymal changes early is essential in differentiating between conditions such as HP and idiopathic interstitial pneumonias [13]. The ground-glass opacities reflect inflammation, while mosaic attenuation is indicative of small airway involvement, which is a hallmark of HP [16]. Early identification of these changes on HRCT can prevent progression to irreversible fibrosis [17].

Management and outcomes

Treatment for BFL is based on two approaches: one is to reduce the inflammation, usually with corticosteroids, and the other is to avoid antigen exposure strictly to prevent recurrence. In our case, oral corticosteroids (prednisolone, 40 mg/day) were given for two weeks and then tapered off over three months [12]. Moreover, the patient was counseled on removing the pigeons from his surroundings and avoiding further exposure to avian antigens [11]. The patient's symptoms resolved within three months of treatment, and follow-up chest X-rays did not reveal evidence of disease progression. Follow-up HRCT performed after six months showed stable lung parenchyma without evidence of fibrosis [13]. The patient remained symptom-free one year after diagnosis because of the strict avoidance of pigeons [11]. This case also underlines the importance of early intervention and environmental control in preventing the progression of HP to chronic forms that may result in permanent lung damage.

Long-term corticosteroid therapy is associated with a number of risks, including metabolic complications such as hyperglycemia and hypertension, as well as osteoporosis and an increased susceptibility to infections [18]. A specific and serious side effect is osteoporosis, and bone density has to be followed regularly and the risk of fracture prevented by supplements of calcium and vitamin D. Long-term use also requires tapering to prevent adrenal insufficiency upon withdrawal after chronic therapy [19]. For safety, regular follow-up should be performed in patients with corticosteroid therapy, including monitoring of glucose and blood pressure, infection, and adrenal function during the reduction of the dose.

Clinical course

Based on the HRCT findings of the patient, a diagnosis of BFL was made that improved within three months of treatment with corticosteroid therapy. During the long follow-up since the diagnosis was made, serial chest X-ray and HRCT at six months and one year showed no instability in lung parenchyma, with no evidence of fibrosis and no recurrence of the disease [15]. Early recognition of characteristic HRCT features at presentation, such as ground-glass opacities and mosaic attenuation patterns typical for HP, played an important role in timely management [16]. Avoidance of bird-related exposures, as advised, was important for the patient to avoid recurrence, hence underlining the importance of environmental control in the management of HP [20].

## Conclusions

This case underlines the importance of distinguishing BFL from other ILDs by obtaining a meticulous history of avian exposure and characteristic HRCT findings of HP such as centrilobular ground-glass opacities and mosaic attenuation and excluding other etiologies such as idiopathic pulmonary fibrosis and sarcoidosis. Recognition of these differentiations allows for early diagnosis and proper treatment, which is very important to improve the outcome of patients.

For clinical practice, this case points out the routine consideration of environmental and occupational exposure in patients with respiratory complaints, early and detailed history-taking about bird contact, and fast application of HRCT to prevent diagnostic delays. Treatment should focus on corticosteroid management, strict antigen avoidance, and long-term follow-up to reduce recurrence and long-term complications. Differentiating BFL from the rest is critical, because treatment options vary greatly and there would be a difference in outcomes. Integration of environmental and occupational health considerations into diagnostic frameworks is central to the optimization of care in ILDs.
